# Visual and Hearing Sensitivity Affect Robot-Based Training for Children Diagnosed With Autism Spectrum Disorder

**DOI:** 10.3389/frobt.2021.748853

**Published:** 2022-01-12

**Authors:** P. Chevalier, D. Ghiglino, F. Floris, T. Priolo, A. Wykowska

**Affiliations:** ^1^ Social Cognition in Human-Robot Interaction, Istituto Italiano di Tecnologia (IIT), Genoa, Italy; ^2^ DIBRIS, Università degli Studi di Genova, Genoa, Italy; ^3^ Piccolo Cottolengo Genovese di Don Orione, Genoa, Italy

**Keywords:** autism, robot, human-robot interaction, robot-assisted therapy, sensory sensitivity

## Abstract

In this paper, we investigate the impact of sensory sensitivity during robot-assisted training for children diagnosed with Autism Spectrum Disorder (ASD). Indeed, user-adaptation for robot-based therapies could help users to focus on the training, and thus improve the benefits of the interactions. Children diagnosed with ASD often suffer from sensory sensitivity, and can show hyper or hypo-reactivity to sensory events, such as reacting strongly or not at all to sounds, movements, or touch. Considering it during robot therapies may improve the overall interaction. In the present study, thirty-four children diagnosed with ASD underwent a joint attention training with the robot Cozmo. The eight session training was embedded in the standard therapy. The children were screened for their sensory sensitivity with the Sensory Profile Checklist Revised. Their social skills were screened before and after the training with the Early Social Communication Scale. We recorded their performance and the amount of feedback they were receiving from the therapist through animations of happy and sad emotions played on the robot. Our results showed that visual and hearing sensitivity influenced the improvements of the skill to initiate joint attention. Also, the therapists of individuals with a high sensitivity to hearing chose to play fewer animations of the robot during the training phase of the robot activity. The animations did not include sounds, but the robot was producing motor noise. These results are supporting the idea that sensory sensitivity of children diagnosed with ASD should be screened prior to engaging the children in robot-assisted therapy.

## 1 Introduction

In Human-Robot Interaction (HRI), and especially in settings where robots serve the role of social assistants, user-adaptation is an important factor to study. Indeed, fitting the behavior of the robot to the user’s needs can improve the social interaction during HRI. During the last decades, inter-individual differences were investigated in HRI [e.g., users’ personality (Robert, 2018)], or their prior experience with robots (Bartneck et al., 2007) to observe how much they may affect the interaction with robots. Similarly, the robot’s specifics were investigated, for example how much robots’ embodiment (Deng et al., 2019) or displayed personality (Mou et al., 2020) can influence the interaction. Understanding inter-individual differences among users would help robot designers to endow artificial agents with features that can smoothen the interaction, making it more engaging for the human counterpart. This aspect is crucial in socially assistive robotics, where tailor-made technical solution could have an impact on the clinical outcome (Khosla et al., 2015; Mataric, 2017; Clabaugh et al., 2019).

To address inter-individual differences among users, sensory sensitivity profiles can be crucial. Sensory sensitivity is defined as the detection and reaction ones can have regarding sensory events (e.g., visual, auditory, touch, taste, smell, vestibular, and proprioception) (Dunn, 1999). Individuals perceive and react differently to sensory information. For example, some individuals would seek a quiet environment to work, when others would turn on the TV or the radio to have background noises. Some would work in a bright environment, whereas others would close the blinds. During social interaction we constantly process sensory information (i.e., the use of vision cues when we look at our interlocutor’s facial expression, or the use of auditory cues when we listen to our interlocutor voice tone). Robots are a complex source of sensory information in a their own way (particular embodiment, presence of mechanical parts, LEDs, or noises from the motor) and can affect the interactions with the user (Deng et al., 2019). A number of studies reported that sensory sensitivity could predict behaviors in both clinical and general (healthy) population (in clinical population: Chevalier et al., 2016; Chevalier et al., 2017; Chevalier et al., 2021a; and in general population: Agrigoroaie and Tapus, 2017; Chevalier et al., 2021b). Sensory sensitivity affects the task performance of healthy adults during a human-robot interaction. The performance in a Stroop task with the Tiago robot (PAL robotics, Pages et al., 2016) was shown to be influenced by the participants’ auditory sensitivity (Agrigoroaie and Tapus, 2017)*.* In a recent work (Chevalier et al., 2021b), the authors investigated the influence of visual sensitivity on the performance on an imitation task with both a physical and virtual version of the R1 robot (Parmiggiani et al., 2017). Results showed that higher visual sensitivity increased imitation accuracy, providing evidence that screening user’s sensory sensitivity is a helpful tool to evaluate and design agent-user interactions. Sensory sensitivity was also investigated in clinical populations. In a series of studies, vision and proprioception sensitivity was found to predict the performance of children diagnosed with Autism Spectrum Disorder (ASD) in imitation (Chevalier et al., 2017) and joint attention (Chevalier et al., 2016) during an interaction with the robot NAO (Softbank robotics, Gouaillier et al., 2008). Furthermore, visual and proprioception sensitivity predict performance in an emotion recognition task that involved two robots (Nao from Softbank Robotics and R25 from Robokind), an avatar (Mary from MARC, Courgeon and Clavel, 2013), and a human agent. In all the above-mentioned studies, participants with visual hyper-reactivity and proprioception hypo-reactivity showed higher performance in all the tasks compared to the participants showing vision hypo-reactivity and hyper-reactivity on proprioception. Finally, in Chevalier et al. (2021a), children diagnosed with ASD played a “Simon says” game with the iCub robot presented on a monitor screen, once with the motor noises turned on, and once with the motor noises turned off. The results showed that participants reporting to be overwhelmed by unexpected loud noises were more able to focus on the game when the robot’s motor noises were turned off.

The findings from these studies show that sensory sensitivity appears to affect the social interactions between a human and a robot in both clinical and general population. Additionally, the sensory stimuli generated by the robot’s embodiment are reported to affect the interactions. Therefore, understanding the effect of sensory sensitivity in HRI, appears to be of great importance, and it will be impactful for both healthy and clinical populations.

This statement is particularly true regarding the clinical population of individuals with ASD. Indeed, this population seems to benefit from the use of socially assistive robots during standard therapy (Aresti-Bartolome and Garcia-Zapirain, 2014; Coeckelbergh et al., 2016; Ghiglino et al., 2021). This might be due to the specificity of impairments of ASD individuals, which include deficient communication and social skills, along with restricted and repetitive patterns of behaviors, interests, or activities (APA, 2013). Robots are believed to be a fitting tool for this population as they present a mechanistic and predictable nature, which appears to be attractive and reassuring for children with ASD (Scassellati et al., 2012; Cabibihan et al., 2013). Children with ASD participating in robot-assisted intervention showed improvements in their social skills or a reduction of repetitive behavior (Scassellati et al., 2012). However, the sensory stimulation due to the robots’ mechanical embodiment and motor noise during interactions designed for children diagnosed with ASD needs to be addressed. Indeed, individuals with ASD suffer also from sensory hypo or hypersensitivity (APA, 2013) and some authors claim that robots are an overwhelming source of sensory stimulation (Ferrari et al., 2009). Thus, even if robots seem to be beneficial for ASD individuals (Scassellati et al., 2012; Pennisi et al., 2016), the attentional engagement they require from the individual might be detrimental for the processing of the social aspect of the interaction (van Straten et al., 2018). Understanding how sensory sensitivity affects HRI would improve the design of tailor-made robot-assisted interventions developed for children with ASD.

The aim of this study is to explore the effect of sensory sensitivity on improvements of social skills related to a joint attention training with the Cozmo robot (Anki Robotics/Digital Dream Labs). The training of joint attention is often included in standard ASD treatment plan, as impairments of joint attention are typical of ASD individuals (Mundy and Newell, 2007; APA, 2013; Mundy, 2018). Joint attention is claimed to be a fundamental prerequisite of mentalizing abilities, as its development allows an individual to share the focus of his/her attention with another individual, attending the same object or event (Emery, 2000). However, children diagnosed with ASD use uncommon joint attention strategies (Charman et al., 1997; Johnson et al., 2007; Mundy and Newell, 2007). The inclusion of joint attention training in the standard treatment plan of ASD showed positive effects on social learning (Johnson et al., 2007; Mundy and Newell, 2007). The use of robot-assisted therapies of ASD tailored on joint attention showed encouraging results [see (Chevalier et al., 2020) for a review].

## 2 Methods

### 2.1 Participants

Thirty-six children diagnosed with ASD were recruited at the Piccolo Cottolengo Genovese di Don Orione (Genoa, Italy) (age = 5.69 years ± 1.06, five females). Prior to the beginning of the study, participants’ formal diagnosis of ASD was confirmed healthcare professionals of Piccolo Cottolengo Genovese di Don Orione, using the ADOS screening tool (Rutter et al., 2012). Parents or legal tutors of the children recruited for the study were asked to provide the healthcare professionals with a signed written informed consent. Our experimental protocols followed the ethical standards laid down in the Declaration of Helsinki and were approved by the local Ethics Committee (Comitato Etico Regione Liguria).

### 2.2 Experimental Design

Our previous paper (Ghiglino et al., 2021) describes in more detail the training protocol and its efficacy. Here, we focus specifically on how the sensory sensitivity of the children, screened with the Sensory Profile Checklist Revised (SPCR, Bogdashina, 2003), affected the interaction with the robot.

**FIGURE 1:**
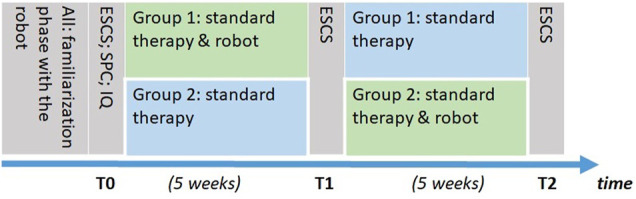
Timeline of the study. First, all participants did the familiarization phase. Participants remaining in the training protocol were separated in two groups. All the remaining participants were then screened for their social skills with the Early Social Communication Scale (ESCS), their sensory sensitivity with the Sensory Profile Checklist Revised (SPCR) and their IQ (Griffiths) at T0. Then, Group 1 did the Standard therapy and robot condition and Group 2 the Standard therapy condition. After 5 weeks, children from both groups were screened with the ESCS at T1. Then, Group 1 did the Standard therapy condition and Group 2 the Standard therapy and robot condition. After 5 weeks, children from both groups were screened with the ESCS at T2.

Due to the limited sample size, and to grant all the children involved in the study the possibility to interact with the robot, we investigated the efficacy of the training using a two-period crossover design. Participants were pseudo-randomly assigned to two groups, balanced by their chronological age and by their ADOS score. Participants in one group (Group 1) received the robot-assisted intervention during the first period of the study, while participants in the other group (Group 2) received it during the second period of the study (see Figure 1). A trained psychologist screened the children’s social skills with the ESCS three times during the study. The first time, before the beginning of the experiment (T0), the second time after the first period of the study (T1), the third time after the second period of the study (T2) (see Figure 1). Additionally, prior to the beginning of the activities, participants’ IQ and sensory sensitivity were screened using the Italian versions of Griffiths’ Developmental Scales (Green et al., 2015) and the Sensory Profile Checklist Revised (SPCR, Bogdashina, 2003).

The activities with the robot were always embedded in the standard therapy. Therefore, children interacted with the Cozmo robot during their 1 h therapy with their usual therapist. Prior to the training activity, all participants underwent a familiarization phase with a simpler version of the training. The familiarization was supposed to ensure that the children involved in the study were all able to understand the instructions of the training, and were at ease with the robot. After the familiarization phase, ten participants were excluded from the experiment, as they were not able to perform the task or were too uncomfortable with the robot. Two participants were withdrawn from the training due to familial reasons. Two participants were excluded as their SPCR evaluation was missing. In total, 22 participants were considered for the analysis in the present study (see Table 1 for details on the groups and participants).

**TABLE 1:** Participants’ details by group.

ADOS levels	Group 1			Group 2		
	Number and gender	Age	IQ (Griffith)	Number and gender	Age	IQ (Griffith)
Level 1	5M	5.6 ± 1.0	78.6 ± 17.0	3M-1F	6.0 ± 0.7	75.0 ± 17.1
Level 2	3M	6.0 ± 0.8	51.3 ± 4.6	3M-1F	6.0 ± 0.7	64.5 ± 12.9
Level 3	2M-1F	5.0 ± 0.8	32.7 ± 11.9	2M	5.5 ± 0.5	46.0 ± 13.0
Total	10M-1F	5.5 ± 1.0	58.6 ± 23.6	8M-2F	5.9 ± 0.7	65.0 ± 18.1

### 2.3 Setup and Training Procedure

We used the robot Cozmo, a low-price commercial toy robot, provided with cubes that can be lit in different colors. We designed a program for Cozmo that enabled the therapists to conduct the experiment autonomously, without the need for an experimenter to support them. The therapists were trained to prepare the setup and launch the experiment. Following Anki’s SDK setup^1^ we developed a custom Python program to control the robot via a webpage. Based on the documentation provided by Anki to use Cozmo with a custom program, the setup required the robot Cozmo, a tablet (here, Android) with the Anki application on SDK mode connected via USB to a computer (here, Windows 10) which ran the Python code. A Python script controlled the training sequence and the robot’s behaviors, and a webpage developed in HTML and JavaScript acted as a user interface. Flask^2^ served as a server to communicate between the Python script and the webpage. The user interface enabled the therapist to navigate through the training, and record the participant’s identifier and correct/incorrect answers.

**FIGURE 2:**
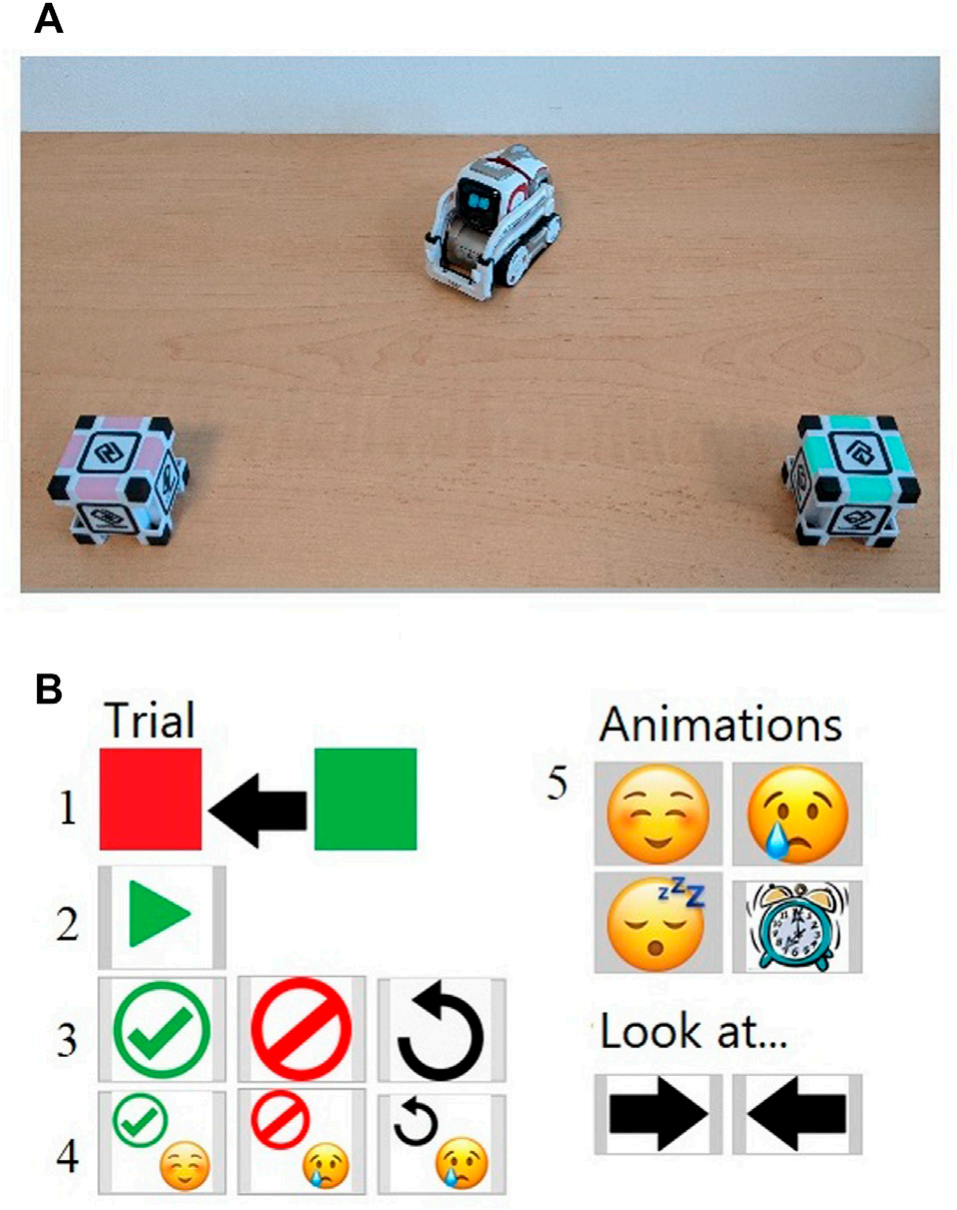
**(A)** The robot and the cubes; **(B)** Interface provided to the therapist. After having clicked on the “play” button (2), the robot put itself in the position as displayed in **(A)**. On the interface **(B)**, the position and the colors of the cubes are reproduced (1). The therapist can enter the response of the child as “correct” or “incorrect” or to repeat the movement if the child was not paying attention. The feedback given to children can be accompanied or not with an animation (respectively in 3 and 4). At the end of the session, the therapist can play some animations on the robot as a reward (5).

The activity with the robot had a duration of 10–15 min, it was conducted once or twice a week (depending on the child’s therapy frequency) during a period of 5 weeks, for a total of eight sessions with the robot per child. The therapists were trained to control the robot in full autonomy (no experimenter was present on-site during the experiment). The robot activity was a joint attention training based on a spatial attention cueing paradigm, with Cozmo delivering twelve joint attention cues. For each trials, the robot turned and gazed to one of the two cubes. Then, the cubes lit in different colors (between red, blue, green and yellow). The therapist asked the child which cube was looking Cozmo, and then recorded the answer of the child thanks to the interface. Finally, the robot went back to its initial position and the cubes turned off. The robot was able to deliver two animations of emotions during the training, happiness and sadness. These were two simple custom animations with a smile or a frowning face appearing on the LED display of Cozmo, and simple body movements (moving the arms ups and down with the face looking up for the happy animation, and looking down with small body movements to the left and right for the sad animation). The therapist chose if the robot performed these animation to reinforce correct or incorrect answer, or at the end of the twelve trials as a reward for participation. The therapist could skip the robot’s animation if she/he considered that they did not provide reinforcement but a distraction (for example, if a child disliked the animations). The therapist was able to restart the trial if the child was not attentive to the robot. Each session contained twelve gaze cueing trials. At the end of the session, the therapist proposed to the child if she/he wanted to see again the animation on the robot before saying to it goodbye, and had the possibility to register some additional comments about the session. Then, standard therapy with the child continued. The full protocol is described in (Ghiglino et al., 2021). Figure 2 depicts the setup and the interface used in the training.

### 2.4 Measurements

#### 2.4.1 Early Social Communication Scale

The ESCS (Seibert and Hogan, 1982) provides measures of individual differences in nonverbal communication skills that emerge typically in children between 8 and 30 months of age. It is a videotaped structured observation measure that requires between 15 and 25 min to administer. The scale evaluates three different nonverbal communication skills: joint attention (subscales: initiating, responding and maintaining joint attention), social interaction (subscales: initiating, responding and maintaining social interaction), and behavioral request (subscales: initiating and responding to behavioral request). ESCS was used as pre and post-test to screen the social skill variations of the children in both conditions (i.e., standard therapy with the robot activity and standard therapy without the robot activity).

#### 2.4.2 Sensory Profile Checklist Revised

The Sensory Profile Checklist Revised (second edition) (Bogdashina, 2003) assesses the individual’s sensitivity in vision, audition, touch, smell, taste, proprioception, and vestibular perception. The questionnaire enables to clarify the strength and weakness of a child regarding their sensory profiles. Each sense is investigated by 20 categories that explore the child sensitivity. The categories assess different aspects of sensory sensitivity, for examples being unable to stop to feel a sensory change (category 2), or acute sensitivity to some stimuli (category 7). For each sense, each category has specific questions (the number of questions in the categories are uneven across sense, for a total of 312 questions) to be answered to evaluate if the child displays the assessed behavior in the category. So, the more a child shows atypical sensory behaviors, the more categories will be marked as true. For each sense, the 20 categories are added to obtain a score from 1 to 20, with a higher score corresponding to a higher sensitivity to the sense. This questionnaire does not take place as a diagnosis tool, but as a help for the parents and therapists to understand the children’s sensory sensibility and therefore adapt their environment and activities (Robinson, 2010). The SPCR was administered by the therapists in charge of the children at the Piccolo Cottolengo Genovese di Don Orione.

#### 2.4.3 Performance Score

Participants’ performance score was computed as the rate of the correct answers in the total of the joint attention trials they performed during the whole training.

#### 2.4.4 Number of Robot’s Animation Played

During the sessions with the robot, the therapist was able to choose to make the robot do the “happy” or “sad” animations during the training. We identified those animations as events that were providing visual and auditory cues to the children. The robot does body movements which can trigger the visual sensitivity, and noises coming from the motor can trigger auditory sensitivity. As the therapist could choose to trigger the robot if they considered it to be beneficial for the child, we considered that it could reflect the sensory sensitivity of the child towards the robot. We recorded how many times the animations were played on the robot during the whole training. Thus, for each participants we collected three measures: the total of animations played during the sessions, the total of animations played as a reinforcement for a correct or incorrect answer, and the total of animations played as a reward for participation.

### 2.5 Data Analysis

We used multiple linear regression analysis to test if the SPCR scores in vision, hearing, touch, and proprioception (we discarded smell, taste, and vestibular perception from our analysis) were predictors of the following dependent variables: the children’s improvements in the ESCS items (for both standard therapy with the robot activity and standard therapy without the robot activity), the children’s performance in the joint attention activity with the robot, and the children’s exposure to the robot’s animations (in total; as reinforcement to an answer; and after the training).

## 3 Results

### 3.1 Children’s Improvements in the ESCS Items

#### 3.1.1 During Standard Therapy With the Robot Activity

**FIGURE 3:**
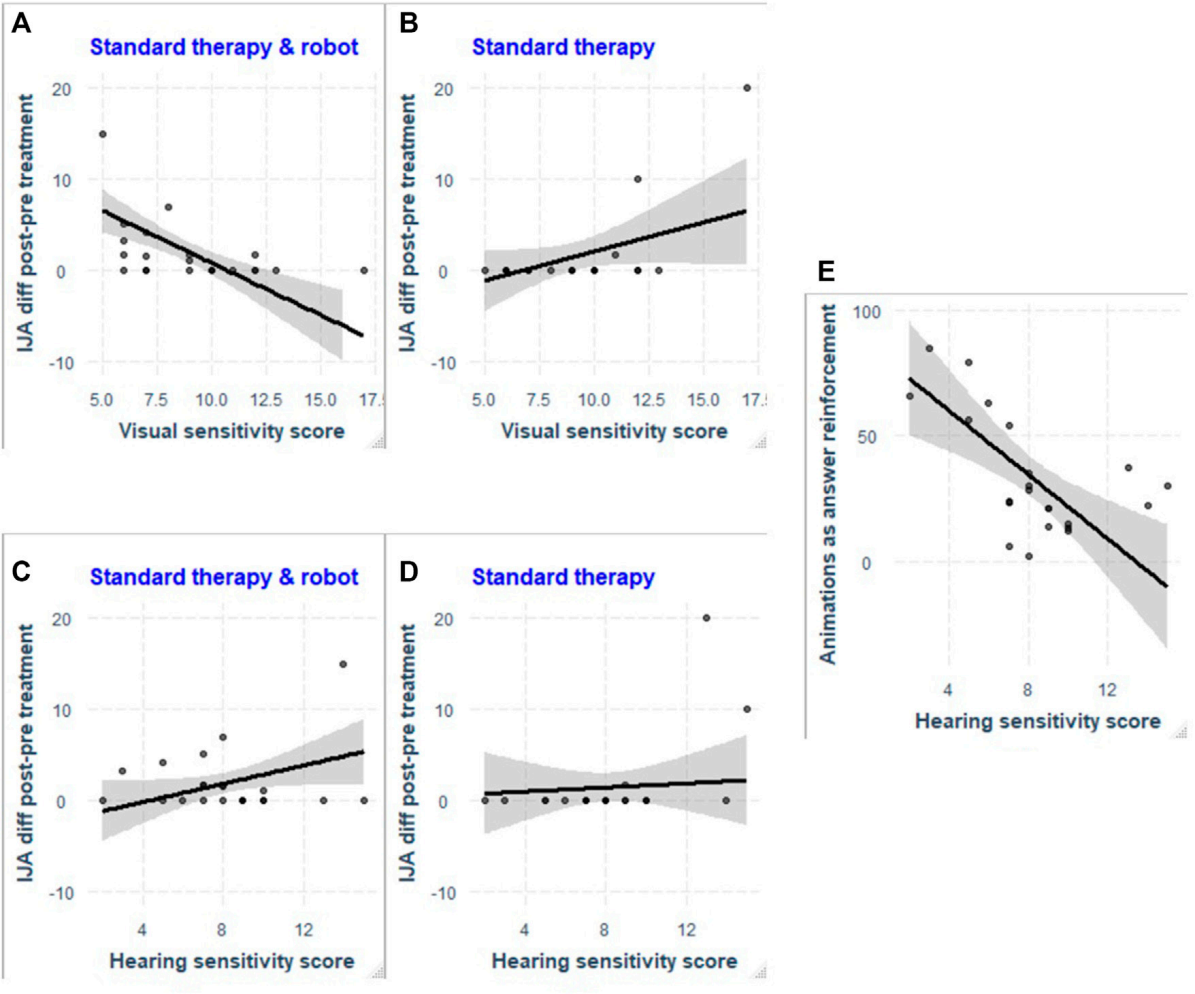
Scatter plot of the difference in scores in the initiating joint attention item of the ESCS as a function of the participants visual sensitivity score, during Standard therapy and robot condition **(A)** and Standard therapy **(B)**; and as a function of the participants’ hearing sensitivity score, during Standard therapy and robot condition **(C)** and Standard therapy **(D)**. The visual and hearing sensitivity scores significantly predicted the improvement in Initiating Joint Attention during Standard therapy and robot condition [**(A)** and **(C)**, respectively], but did not in the Standard therapy alone [**(B)** and **(D)**, respectively]. In **(E)**, the scatter plot of the number of animation played as a reinforcement to a correct or an incorrect answer in function of the hearing sensitivity of the participants.

We found a significant regression for *Initiating Joint Attention* item of the ESCS with the SPCR scores as predictors during the standard therapy augmented by the robot activity (*R2* = 0.584, *F* (4,17)= 5.965, *p* < 0.01). We found that the visual and hearing sensitivity scores significantly predicted the improvement in Initiating Joint Attention (Visual: *β* = −1.14, *p* < 0.001; Hearing: *β* = 0.5, *p* < 0.05). See Figures 3A,C. We did not find other significant regressions between the sensory sensitivity and the improvements of the ESCS items during standard therapy augmented by the robot activity.

#### 3.1.2 Standard Therapy Without the Robot Activity

During the standard therapy without the robot activity, we found a significant regression for the *Initiating Joint Attention* item of the ESCS with the SPCR scores as predictors (*R2* = 0.557, *F*(4,17)= 5.34, *p* < 0.01). However, none of the factors included in the model predicted the performance. See Figures 3B,D where we reported the plots for both hearing and visual sensitivity, for comparison for the standard therapy with the robot activity. We did not find other significant regressions between the sensory sensitivity and the improvements in the ESCS items during standard therapy without the robot activity.

### 3.2 Children’s Performance to the Joint Attention Activity

We did not find any significant regressions between the SPCR scores and the performance of the children in the joint attention task.

### 3.3 Number of Animations Played

We found a significant regression for the total of animation played as a reinforcement for a correct or incorrect answer and the participants’ SPCR scores (*R2* = 0.557, *F*(4,17) = 5.35, *p* < 0.01). We found that hearing sensitivity significantly predicted the number of animations played as answer reinforcement to a correct or incorrect answer (*β* = −6.39, *p* < 0.01) (see Figure 3E). We did not find significant relationships between the sensory sensitivity and the total number of animations played or the total of animation played at the end of the sessions.

## 4 Discussion

In this study, we investigated how sensory sensitivity can affect robot-assisted therapy for children with ASD. Individuals’ visual and auditory sensitivity seem to affect the clinical improvement in *initiating joint attention* when the therapy included the activity with the robot. More precisely, children with ASD with low sensitivity to vision improved better in their *initiating joint attention* skill than the participant with higher vision sensitivity. We can speculate that participants with lower sensitivity to vision process social cues from the robot with ease. Importantly, sensitivity to vision did not affect the clinical outcome of the standard therapy alone. The opposite pattern of results was found when we considered sensitivity to hearing as predictor of the clinical outcome. Specifically, children with ASD that were more sensitive to hearing improved better in their *initiating joint attention* skill than the children with lower hearing sensitivity, when the standard therapy was combined with the activity with the robot. We hypothesize that participants highly sensitive to hearing could be more sensitive to motor sounds, provided by the robot during the training. Similarly as in the visual sensitivity, the hearing sensitivity did not affect the clinical outcome of the standard therapy alone. From these results, we have elements to believe that the children’s improvements in their social skills during such type of interventions would benefit from different behaviors of the robot. Future experiments assessing different robot behaviors would allow for a proper evaluation of user’s need. For example, for the visual sensitivity, we can speculate that participants with higher visual sensitivity could benefit from a robot that shows lower social cues at the beginning of the training, progressively increasing the complexity of social cues during the course of the training (e.g., starting with a robot that shows simple animations in terms of body behavior or facial expressions, and gradually moves towards more complex behaviors and expressions). Similarly, for the hearing sensitivity, we can hypothesize that participants with higher sensory sensitivity could beneficiate from a quieter robot [see (Chevalier et al., 2021a) for similar results]. Robots designed for individuals with high sensitivity could, for example, perform behaviors with a lower number of movements, or movements involving quieter actuators to fit the user-preferences.

Interestingly, the effect of sensory sensitivity was observed on a skill that was not directly addressed during the activity with the robot. Indeed, sensory sensitivity predicted the improvement of Initiating Joint Attention, whereas the Response to Joint Attention was trained in this protocol. Accordingly to the ESCS, responding to joint attention refers to the ability to follow the direction of gaze and gestures of others whereas initiating joint attention refers to the ability to use direction of gaze and gestures to direct the attention of others to spontaneously share experiences (Seibert and Hogan, 1982). Following these definitions, the training with the robot was focused on responding to joint attention, as the children had to follow the movement of the robot to know which color the robot looked at. Variation in the Initiating joint attention skill has been discussed to be driven by social motivation by Mundy et al. (2009), with infants showing more interest in social events and engage more in initiating joint attention. We speculate that the improvement of this skill predicted by the visual and auditory sensitivity may be driven by an increase interest and motivation during the interaction with the robot, and beneficiated the children during interactions with an adult. Participants’ performance in the task was not found to be linked to their sensory sensitivity, in contrast to the results obtained in (Chevalier et al., 2016).

We also observed that the participants with higher hearing sensitivity were exposed to less animation during the joint attention trials, compared to individuals with less sensitivity. The children’s therapists, who were in charge of activating or not the animations, seemed to avoid providing positive or negative feedbacks though the robot to the children with higher hearing sensitivity. However, the same pattern was not observed when the therapist had to display the conclusive animation of the robot when the training session was completed. This result suggests that children that have a high sensitivity in hearing might have preferred fewer animations from the robot, meaning less noise from the robot’s motors, during the training phase. However, as the animations were regulated by the therapist, there may have been other reasons not to play them during the training. The therapist did not report the reason why they played (or not) the animations during the training phase.

Future works aimed at adapting robot interventions to the sensory sensitivity of children diagnosed with ASD could implement one of the following strategies. The first one would be to assess sensory sensitivity with a questionnaire (such as the Sensory Profile Checklist Revised, Bogdashina, 2003; the Short Sensory Profiles, Tomchek and Dunn, 2007; the Sensory Profiles, Dunn, 2009) before doing the interaction and to “manually” adapt the interaction to the child. A second one would be to study if the sensory sensitivity could be measured using machine learning techniques, via computer vision for example, tracking events as avoiding behaviors (e.g., covering the ears when a motor sound occurs) or seeking behaviors (e.g., engaged with the robot during animations).

As final words, this work suggests that sensory sensitivity has an impact on the outcomes of the robot-assisted therapy, and therefore, encourages pursuing research in this direction. Therefore, the animations implemented in assistive robots as a reward during therapeutic activities with ASD individuals should include different levels of intensity that can be adapted to the individual’s sensitivity.

## Data Availability

The raw data supporting the conclusion of this article will be made available by the authors, without undue reservation.

## Footnotes

1 see http://cozmosdk.anki.com/docs/index.html.

2
https://flask.palletsprojects.com/en/1.1.x/.
